# HIFU-CCL19/21 Axis Enhances Dendritic Cell Vaccine Efficacy in the Tumor Microenvironment

**DOI:** 10.3390/pharmaceutics17010065

**Published:** 2025-01-06

**Authors:** Bum-Seo Baek, Hyunmi Park, Ji-Woong Choi, Eun-Young Lee, Seung-Yong Seong

**Affiliations:** 1 Wide River Institute of Immunology, Seoul National University College of Medicine, Hongcheon 25159, Gangwon, Republic of Korea; bumseobaik442@gmail.com (B.-S.B.); phm1721@gmail.com (H.P.); zenith2@snu.ac.kr (J.-W.C.); ley3016@snu.ac.kr (E.-Y.L.); 2 Department of Microbiology and Immunology, Seoul National University College of Medicine, Seoul 03080, Republic of Korea; 3 Department of Biomedical Sciences, Seoul National University College of Medicine, Seoul 03080, Republic of Korea; 4 Shaperon Inc., Ltd., Seoul 06373, Republic of Korea

**Keywords:** mechanical high-intensity focused ultrasound, tumor microenvironment, cancer immunotherapy, dendritic cell vaccines

## Abstract

Background/Objectives: Effectively targeting treatment-resistant tumor cells, particularly cancer stem cells (CSCs) involved in tumor recurrence, remains a major challenge in immunotherapy. This study examines the potential of combining mechanical high-intensity focused ultrasound (M-HIFU) with dendritic cell (DC) vaccines to enhance immune responses against OLFM4-expressing tumors, a CSC marker linked to immune evasion and tumor growth. Methods: M-HIFU was applied to induce immunogenic cell death by mechanically disrupting tumor cells, releasing tumor-associated antigens and creating an immunostimulatory environment. DC vaccines loaded with OLFM4 were then administered to boost the immune response within this primed environment. Results: The combination of M-HIFU and DC vaccine significantly inhibited tumor growth and metastasis, with enhanced T-cell activation and increased recruitment of immune cells due to elevated chemokines CCL19 and CCL21. This synergy promoted immune memory, reducing the likelihood of recurrence. Conclusions: M-HIFU effectively promotes the migration of DC vaccines through CCL19/21, presenting a promising approach for cancer treatment. Further studies are recommended to optimize this combination for clinical applications, with potential to improve patient outcomes in challenging cancer types.

## 1. Introduction

Traditionally, cancer therapy has relied on approaches such as surgery, chemotherapy, and radiation, aimed at directly removing or destroying tumors. While these methods have effectively treated many cancer types, they often fall short in aggressive cancers due to the emergence of treatment-resistant tumor cells. Cancer stem cells (CSCs), with their self-renewal capabilities and pivotal roles in tumor recurrence and metastasis, present a major challenge in cancer therapy [1]. These cells commonly resist standard treatments, leading to relapse even after the primary tumor has been eliminated. To address this, a multifaceted approach is needed to target both the tumor mass and CSCs, which are central to treatment resistance.

Immunotherapy has garnered significant attention as a strategy to overcome the limitations of traditional cancer treatments. By activating the body’s immune system to target cancer cells, immunotherapy provides a more precise approach, particularly effective when other treatments fall short [2]. Among the most promising immunotherapeutic strategies are dendritic cell (DC) vaccines. Dendritic cells, as primary antigen-presenting cells, uniquely bridge innate and adaptive immune responses, facilitating T-cell recognition of cancer cells [3]. By loading DCs with tumor-associated antigens, these vaccines prime the immune system to mount a robust and targeted attack on cancer cells, especially those capable of evading conventional therapies.

A major advancement in DC vaccine research is the targeting of CSCs, which drive tumor relapse. CSCs express specific markers, such as OLFM4, that distinguish them from the bulk of tumor and normal cells [4].

OLFM4 is overexpressed in many tumor tissues compared to normal counterparts, making it an attractive target for immunotherapy [5,6,7]. In the Melanoma B16F10 model, OLFM4 has also been reported to play a role in tumor progression and immune evasion mechanisms. This suggests its potential as a therapeutic target in melanoma, further emphasizing its broad relevance in cancer immunotherapy [8,9]. Notably, the prognostic implications of OLFM4 vary by cancer type; for instance, reduced OLFM4 expression correlates with prostate cancer progression [7], whereas elevated expression is associated with poorer outcomes in pancreatic cancer [5]. This underscores the importance of understanding the tissue-specific roles of OLFM4 in CSC biology before designing immunotherapeutic strategies.

Our previous report confirmed this finding [10]. OLFM4, a glycoprotein linked to various cancers, enhances CSC stemness and survival, making it an attractive target for DC vaccines [11,12]. Studies have shown that DC vaccines loaded with OLFM4 can induce a potent immune response specifically against CSCs, thereby reducing tumor recurrence and metastasis [13]. This approach holds particular promise when tissue-specific characteristics of OLFM4+ cancers are identified prior to initiating immunotherapy [6]. Additionally, elucidating the complex interplay between OLFM4+ CSCs and immune cells within the tumor microenvironment is crucial for tailoring DC vaccine strategies to maximize their efficacy [5].

While DC vaccines provide a targeted approach to cancer therapy, their efficacy can be further enhanced when combined with other treatments. One such complementary method is high-intensity focused ultrasound (HIFU), a noninvasive technique that uses focused ultrasound waves to ablate tumors [14]. HIFU operates through two primary mechanisms: thermal (T-HIFU) and mechanical (M-HIFU). T-HIFU increases temperature within the target area, resulting in protein denaturation and coagulative necrosis to destroy tumor cells. In contrast, M-HIFU uses lower energy to cause mechanical disruption of tumor cells through cavitation, where microbubbles within the tissue burst and induce cellular damage [15].

M-HIFU has attracted interest in immunotherapy due to its capacity to elicit a robust inflammatory response at tumor sites. Unlike T-HIFU, which relies on heat to kill cells, M-HIFU causes mechanical damage that exposes concealed tumor antigens, creating an immune-stimulatory environment [16]. This process is particularly beneficial for targeting OLFM4+ CSCs, as it reveals tumor antigens critical for enhancing the efficacy of DC vaccines [6]. Furthermore, M-HIFU-induced modifications in the tumor microenvironment can amplify the anti-tumoral effect targeting OLFM4+ CSCs.[5]. This process, known as immunogenic cell death, releases damage-associated molecular patterns that attract immune cells to the tumor site, thereby amplifying the overall immune response. Additionally, M-HIFU’s ability to modify the tumor microenvironment without significant heat damage makes it an ideal partner for immunotherapy approaches, such as DC vaccines, which depend on tumor antigen presentation to activate T cells [17].

When combined, DC vaccines and HIFU produce a synergistic effect that enhances the immune system’s capacity to target and eliminate cancer cells. DC vaccines introduce specific tumor antigens to the immune system, and their effectiveness is further boosted by the immunostimulatory impact of M-HIFU [18]. M-HIFU’s ability to reveal additional tumor antigens, especially those concealed within the tumor microenvironment, complements the antigen-presenting function of dendritic cells. This combined strategy not only increases the variety of antigens available for immune recognition but also strengthens the overall immune response, resulting in more effective elimination of both the primary tumor mass and CSCs [19].

Moreover, the localized inflammatory response induced by M-HIFU promotes the recruitment of immune cells, including dendritic cells, to the tumor site, where they can more effectively process and present tumor antigens [20]. This enhances the efficacy of DC vaccines by ensuring a greater number of immune cells are actively engaged in tumor destruction. The immune system’s ability to recognize and retain these antigens also fosters long-term immune memory, which is essential for preventing tumor recurrence [21,22].

In addition to boosting immune response, the combination of DC vaccines and HIFU minimizes off-target effects. Traditional cancer therapies often damage healthy tissues; however, both DC vaccines and HIFU are highly targeted. HIFU’s focused ultrasound beams precisely deliver energy to the tumor site, sparing surrounding healthy tissues [14,23]. This precision, combined with the specific immune response generated by DC vaccines, offers a treatment strategy with fewer side effects than conventional therapies like chemotherapy and radiation.

Furthermore, molecular analyses of the tumor microenvironment post-HIFU treatment reveal changes in cytokine and chemokine profiles, which are crucial for immune cell recruitment and activation [24]. For instance, cytokines such as IL-12 enhance T-cell activity [25], while chemokines like CCL19 and CCL21 promote dendritic cell migration to the tumor site [26]. Conversely, CCL5 is associated with regulatory T-cell (Treg) recruitment, potentially facilitating immune evasion [27]. However, this study primarily focuses on the role of CCL19 and CCL21 in regulating DC migration and activation rather than the Treg-CCL5 axis. This emphasis underscores the significance of CCL19/CCL21 in optimizing the synergy between DC vaccines and M-HIFU, providing a critical mechanism for precise control over the immune response [28].

In conclusion, combining DC vaccines with HIFU offers a comprehensive and multifaceted approach to cancer therapy. By leveraging the immune-priming effects of DC vaccines alongside the tumor-disrupting capabilities of HIFU, this strategy not only boosts immediate treatment efficacy but also supports long-term immune memory, thereby reducing the risk of tumor recurrence. This approach effectively targets both the primary tumor mass and resistant CSCs, presenting a more sustainable and effective solution for cancer treatment [11]. Further studies on this combination therapy hold the potential to transform cancer treatment, offering hope for improved patient outcomes and enhanced long-term survival rates. In particular, targeting OLFM4+ CSCs through the synergistic use of DC vaccines and M-HIFU represents a critical step toward personalized and precise cancer immunotherapy [5,6,7].

## 2. Materials and Methods

### 2.1. Tumor-Bearing Mouse Models

#### 2.1.1. Mice

C57BL/6 mice (6–8 weeks old) were acquired from the Center for Animal Resource Development at Seoul National University College of Medicine, Republic of Korea. All animal procedures were approved by the Seoul National University (SNU) Animal Welfare Committee under IACUC protocol number SNU-140707-1-1. B6.SJL-PtprcaPepcb/BoyJ mice, also referred to as CD45.1 or Ly5.1, were obtained from The Jackson Laboratory. Animal experiments were performed with the approval of the SNU Animal Welfare Committee under IACUC protocol number SNU-181127-16.

#### 2.1.2. Genes for Recombinant Proteins and Purification of Recombinant Proteins

The design and construction of plasmids encoding human OLFM4 and the production and purification of recombinant OLFM4 proteins were performed following previously published methods [10] Briefly, plasmids encoding the N-terminal fragments of human OLFM4 (nt1084-1626, GenBank accession No. NM_006418.4) were cloned into a pET-28a(+) vector containing a penetratin sequence.

#### 2.1.3. Melanoma Cell Lines Expressing OLFM4

The B16F10 mouse melanoma cell line expressing OLFM4 was established as previously described [10].

### 2.2. Cancer Treatment Strategies

#### 2.2.1. Tumor-Bearing Mouse Models and Immunization

To establish a tumor-bearing mouse model, 6-week-old B6 mice were injected with 2 × 10^6^ B16F10-luc2-OLFM4 cells in the right flank on day 0. The mice were then immunized with DCs pulsed with P-OLFM4 in complete RPMI 1640 medium, as shown in Figure S5. Lung metastases were induced by administering 5 × 10^5^ B16-Luc2-OLFM4 cells in 100 μL of PBS (lacking Ca2^+^ and Mg2^+^) intravenously into the tail vein on day 0. Subsequently, the mice were vaccinated with DC vaccines, which were delivered subcutaneously (S.C.) at the base of the tail or intratumorally (I.T.) at the site of the S.C.-injected tumor.

#### 2.2.2. In Vitro Generation of Bone Marrow-Derived DCs

Bone marrow-derived DCs were generated according to previously established protocols [10]. Briefly, bone marrow cells were harvested from the femurs and tibia of 6- to 10-week-old C57BL/6 mice and cultured in complete RPMI 1640 medium supplemented with 10 ng/mL GM-CSF, 10 ng/mL IL-4, and other additives. DC differentiation was confirmed using flow cytometry (Figure S2) on day 6.

#### 2.2.3. Thermal and Mechanical High-Intensity Focused Ultrasound

Thermal and mechanical HIFU treatments were administered to achieve full tumor coverage while minimizing off-target damage to surrounding tissues. T-HIFU was performed using a high-speed bipolar amplifier (DC~10 MHz, 142V p-p), a multifunction generator (WF1974, NF Inc., Wheaton, IL, USA), an amplifier (HSA4011, NF Inc., Wheaton, IL, USA), an oscilloscope (DPO2024B, Tektronix, IL, USA), and a focused ultrasonic transducer (∅100 mm, focus depth 80 mm) with an impedance matching circuit (50 ohms @ 1 MHz). The treatment protocol consisted of 10 min of application, alternating between 20 s on and 40 s off, to mitigate the risk of overheating. Instead of thermal ablation, M-HIFU was applied using the same setup with a 5% duty cycle to induce cavitation effects. Both HIFU modalities used a focal area of less than 3 mm, and the focal point was systematically shifted in 3 mm increments to ensure comprehensive coverage of the tumor without overlap.

### 2.3. Quantitative Evaluation of Anti-Tumor Immune Responses

#### 2.3.1. Analysis of Tumor Growth

Control mice were treated with PBS (N.C.). Tumor volume was measured every three days using vernier calipers (Mitutoyo) and calculated using the following formula: V = (W^2^ × L)/2, where W and L represent the width and length of the tumor, respectively. Tumor growth was monitored in vivo using bioluminescence imaging with an IVIS imaging system (PerkinElmer). On day 22, the mice were intraperitoneally injected with d-luciferin (150 mg/kg) before whole-body imaging using the IVIS Spectrum-CT in vivo imaging system (PerkinElmer, Waltham, MA, USA). Images were captured 10 min after injection, and signal intensities were quantified using Living Image software V4.0 (PerkinElmer).

#### 2.3.2. Histological Analysis of Lung Tissues

Lung tissues were fixed in 10% formalin, embedded in paraffin, deparaffinized, rehydrated, and stained with hematoxylin and eosin (H&E). For H&E staining, the slides were deparaffinized in xylene (5 min, three times), hydrated through graded ethanol (100%, 95%, 80%, and 70%) for 2 min each, and rinsed in distilled water. Hematoxylin staining (3–5 min) was followed by rinsing with distilled water (5 min), differentiation in 1% acid alcohol, and bluing in aqueous ammonia or lithium carbonate (30 s). After rinsing in distilled water for 5 min, the slides were stained with eosin for 1–2 min, followed by a final rinse in distilled water to complete the staining process.

#### 2.3.3. Lymphocyte Proliferation Assay

Cells isolated from inguinal lymph nodes were labeled with 5(6)-carboxyfluorescein diacetate N-succinimidyl ester (Invitrogen, Carlsbad, CA, USA). For CFSE staining, the cell suspension was incubated with 1 μM CFSE at 37 °C in the dark for 10 min and then quenched with ice-cold fetal bovine serum (FBS) to twice the volume of the cell suspension. The cells were thoroughly washed to remove excess CFSE and cultured for three days in RPMI 1640 medium containing 10% heat-inactivated FBS, 50 nM β-mercaptoethanol (β-ME), 50 μg/mL gentamicin (Invitrogen), 100 U/mL penicillin-streptomycin, and 10 μg/mL purified recombinant OLFM4 protein. After three days, the cells were stained with anti-CD3 (BioLegend, clone 145-2C11) and anti-CD8 (BD, clone 53-6.7) antibodies, and the CFSE dilution in CD3^+^CD8^+^ T cells was analyzed (Figure S3) using FACS LSR Fortessa (BD Biosciences, Franklin Lakes, NJ, USA).

#### 2.3.4. IFN-γ ELISPOT

ELISPOT plates (Millipore, Burlington, MA, USA) were initially coated with an anti-mouse IFN-γ antibody (BD Biosciences, Franklin Lakes, NJ, USA) and blocked with a complete RPMI 1640 medium. Splenocytes (5 × 10^5^ cells/well) were then plated and incubated with 10 μg/mL OLFM4 protein in the pre-coated ELISPOT plates. After three days of incubation, the plates were washed and stained. For staining, the plates were treated with biotinylated anti-IFN-γ antibody (5 μg/mL, BD Biosciences, Franklin Lakes, NJ, USA) at 4 °C for 24 h. The plates were then washed with PBS, and streptavidin-alkaline phosphatase (BD Biosciences, Franklin Lakes, NJ, USA) was added, followed by a 2 h incubation at room temperature. The plates were then washed six times, and BCIP/NBT substrate (Sigma–Aldrich, St. Louis, MI, USA) was added for 30-minute incubation at room temperature. Finally, the plates were washed under running tap water and left to dry overnight at room temperature. The resulting colored spots were counted using an automated image analysis system equipped with an ELISPOT reader (AID GmbH, Penzberg, Germany).

#### 2.3.5. OLFM4-Specific Cytotoxicity Assays

After treatment, the mice were sacrificed, and cells from the inguinal lymph nodes were harvested to assess OLFM4-specific cytotoxic activity. Target cells (B16F10-Luc2-OLFM4) were stained with 5 μM CFSE (CFSE^high^), whereas control cells (B16F10-Luc2-mock) were labeled with 0.5 μM CFSE (CFSE^low^). Lymph node cells were cultured with OLFM4 (10 μg/mL) for three days in vitro to expand the effector cell population. CFSE^high^ and CFSE^low^ cells were mixed at a 1:1 ratio to form responder cells. Effector and responder cells were co-cultured at ratios of 40:1, 20:1, and 10:1. After 16 h of incubation, CFSE dilution in responder cells was analyzed using FACS on an LSR Fortessa cytometer (BD Biosciences), with live cells gated based on DAPI staining. Specific lysis (%) was calculated as 100 × (1 − (% CFSE^high^/% CFSE^low^)).

#### 2.3.6. Dendritic Cell Injection and Migration Analysis via Flow Cytometry

Freshly enriched CD45.1^+^ DCs were resuspended at a concentration of 1 × 10^6^ cells in 100 µL of PBS and injected either S.C. or I.T. into B16F10-Luc-OLFM4 tumors established in C57BL/6 mice. Two days after the final injection, tumor-draining inguinal lymph nodes were collected to assess the migration of the injected DCs. Flow cytometry was used to evaluate the migration of DCs. The inguinal lymph nodes were processed, and total cell counts were obtained using a cytometer cell counter. The cells were then stained with antibodies against CD45.1 (BioLegend, Clone A20) and CD45.2 (BioLegend, Clone 104) to differentiate the injected DCs (CD45.1^+^) from the host cells (CD45.2^+^). Flow cytometry was performed using an LSR Fortessa, and the data were analyzed using FlowJo to determine the percentage of CD45.1^+^ cells in the lymph nodes. Migration efficiency was calculated by dividing the absolute number of CD45.1^+^ cells in the lymph nodes by the total number of injected DCs. Additionally, CCR7 expression in CD45.1^+^ DCs, which is essential for lymph node homing, was analyzed via flow cytometry using anti-CCR7 antibodies (BioLegend, Clone 4B12).

#### 2.3.7. Quantification of CCL19 and CCL21 Levels

The tumors were homogenized in PBS (containing a protease inhibitor cocktail) at a ratio of 1 mL of PBS per 100 mg of tumor tissue. The samples were placed on ice and sonicated to break down the tissues. After homogenization, the sample was centrifuged at 500× *g* for 10 min to pellet the tissue, and the supernatant was collected for ELISA. The levels of the chemokines CCL19 and CCL21 in the tumor microenvironment were quantified using ELISA DuoSet kits (R&D Systems, Minneapolis, MN, USA), following the manufacturer’s protocol. Briefly, 96-well plates were coated overnight with the capture antibody (2 μg/mL per well) provided in the kit, followed by blocking with 1% BSA for 1 h at room temperature. After washing, samples and standards were added to the wells and incubated for 2 h at room temperature. Next, the detection antibody (200 ng/mL per well) was added and incubated for 2 h at room temperature, followed by the sequential addition of streptavidin-HRP. All steps were interspersed with three washes using 0.05% Tween 20. The color reaction was developed using a TMB substrate and stopped with 2N H_2_SO_4_. Absorbance was measured at 450 nm using a microplate reader, and chemokine concentrations were determined based on a standard curve.

#### 2.3.8. Statistical Analysis

Statistical significance was determined using Student’s *t*-test for comparisons between two groups, and one-way ANOVA followed by Tukey’s multiple comparison test for tumor size analysis, performed using SigmaPlot software V12 (Jandel, San Rafael, CA, USA). Data are presented as mean ± standard error of the mean, and a *p*-value of less than 0.05 was considered statistically significant.

## 3. Results

### 3.1. OLFM4-Specific Immune Responses Induced by Mechanical HIFU Compared to Thermal HIFU in Tumor-Bearing Mice

**Figure 1:**
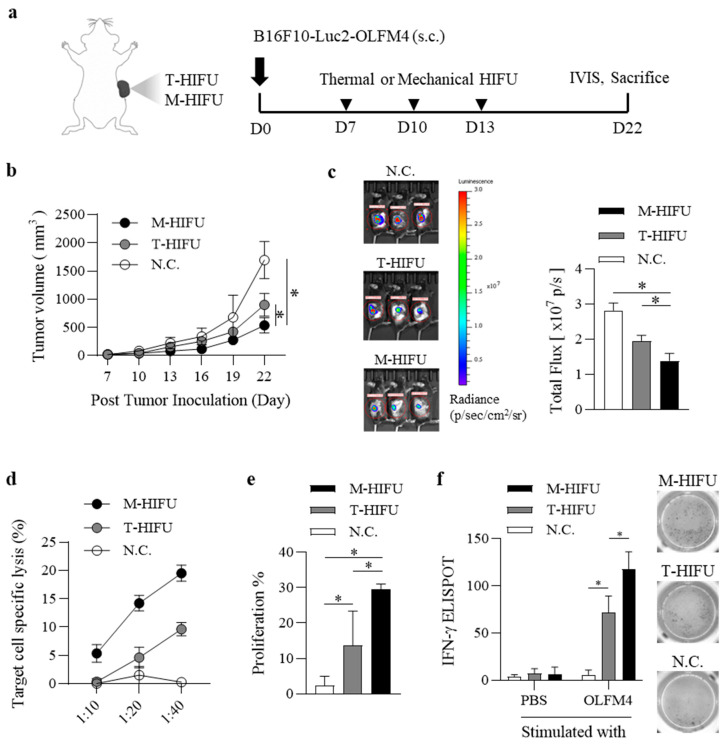
OLFM4-specific immune responses induced by mechanical HIFU compared to thermal HIFU in tumor-bearing Mice. (**a**) Schematic illustrating the timeline of thermal and mechanical HIFU (▼) treatments following inoculation with B16F10-Luc2-OLFM4 tumor cells on day 0. (**b**) Tumor growth monitoring: B6 mice (n = 5/group) were injected with 2 × 10^6^ B16F10-Luc-OLFM4 tumor cells in the right flank 7 days before the first immunization. Tumor size was measured every three days for 22 days. Data are presented as mean ± standard error for 5 mice. (**c**) Luminescence from the tumors was measured using IVIS (**left**), and total flux was quantified (**right**). (**d**) Effector cells were collected 9 days after the final immunization and incubated with CFSE-labeled target cells at an effector-to-target (E:T) ratio of 40:1 for 16 h. The percentage of specific lysis was calculated using the following formula: Specific Lysis (%) = [1 − (% CFSE^high^/% CFSE^low^)] × 100. Data from three independent experiments are shown as mean ± standard error. (**e**) Lymphocytes were collected from inguinal lymph nodes 9 days after the last immunization, stained with CFSE, and co-cultured with PBS or OLFM4 for two days. Lymphocyte proliferation was assessed by CFSE dilution. Representative data from three independent experiments are shown. (**f**) The number of IFN-γ-producing splenocytes was compared by ELISPOT after stimulation with PBS or OLFM4 for two days. * Statistically significant at *p* < 0.05, according to Student’s *t*-test.

We evaluated tumor growth in mice following treatment with T-HIFU and M-HIFU. B6 mice were injected with B16F10-Luc2-OLFM4 cells into their right flanks and received HIFU treatment three times (▼) (Figure 1a). Mice treated with M-HIFU showed a significant delay in tumor growth compared to those treated with T-HIFU (Figure 1b). Luciferase signals from the tumor cells were monitored using an IVIS system (Figure 1c). M-HIFU treatment resulted in a marked tumor growth inhibition of 50.7 ± 14.7% compared to the N.C. group (Figure 1c, right panel), whereas mice treated with T-HIFU showed a 30.7 ± 11.42% reduction in total flux compared to the N.C. group (Figure 1c, right panel).

After HIFU treatment, cytotoxicity of OLFM4-specific lymphocytes (CTLs) in mice was assessed. OLFM4+ target cells and OLFM4-mock cells were labeled with 5 μM CFSE (CFSE^high^) and 0.5 μM CFSE (CFSE^low^), respectively (Figure S1). OLFM4-specific cytotoxicity of cells from the inguinal lymph nodes was evaluated using an S.C. tumor model. In this model, lymphocytes from mice treated with M-HIFU showed increased lysis of OLFM4+ target cells compared to those from mice treated with T-HIFU or N.C. (Figure 1d). The target cell-specific lysis by CTLs in the M-HIFU-treated group increased with the effector-to-target (E:T) ratios 10:1 (5.3 ± 1.27), 20:1 (14.2 ± 1.11), and 40:1 (19.5 ± 1.16) (Figure 1d). Lymphocytes collected from inguinal lymph nodes on day 22 were analyzed for OLFM4-specific proliferation via CFSE staining. Notably, CD8^+^ T cells from M-HIFU-treated mice showed strong proliferation in response to OLFM4 in vitro. In contrast, CD8^+^ T cells from mice treated with T-HIFU or N.C. demonstrated a much weaker response to OLFM4 stimulation than those from the M-HIFU group (Figure 1e). The number of IFN-γ-producing splenocytes was measured by ELISPOT following HIFU treatment. In the subcutaneous tumor model, mice treated with M-HIFU had a significantly higher number of OLFM4-specific IFN-γ-producing splenocytes than those treated with T-HIFU or N.C. (Figure 1f). These results suggest that M-HIFU induces a stronger OLFM4-specific immune response compared to T-HIFU in tumor-bearing mice. The enhanced anti-tumor effects observed with M-HIFU, including increased OLFM4-specific cytotoxic lymphocyte activity and a higher frequency of IFN-γ-producing splenocytes, indicate its potential as a more effective approach for targeting OLFM4-expressing tumors.

### 3.2. Mechanical HIFU Combined with DC Vaccine Therapy for Enhanced Tumor Suppression in OLFM4-Expressing Tumor Models

**Figure 2:**
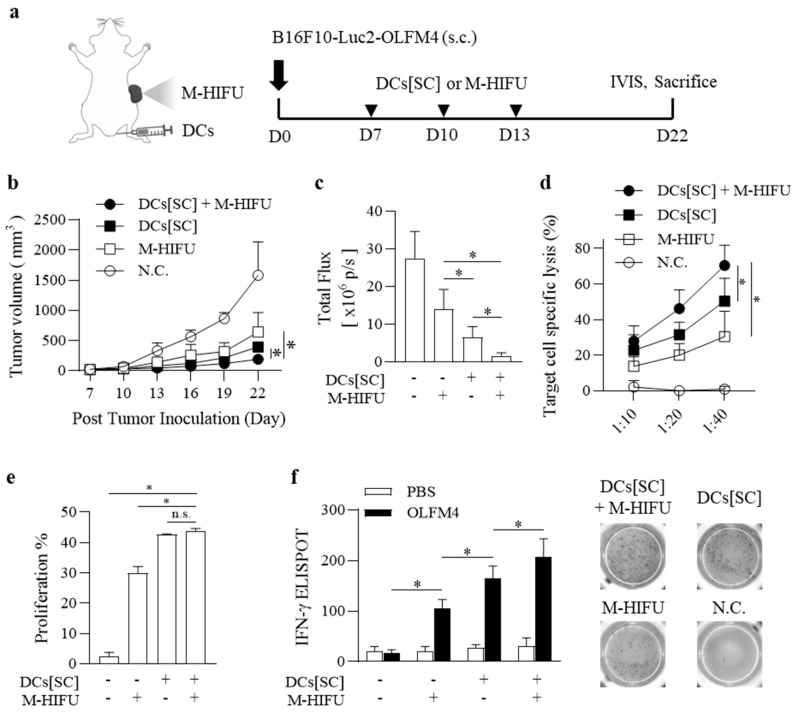
Mechanical HIFU combined with DC vaccine therapy for enhanced tumor suppression in OLFM4-expressing tumor Models. (**a**) Schematic illustrating the timeline of M-HIFU and DCs[SC] treatments (▼) following inoculation with B16F10-Luc2-OLFM4 tumor cells on day 0. (**b**) B6 mice (n = 5/group) were injected with 2 × 10^6^ B16F10-Luc-OLFM4 tumor cells into the right flank 7 days before the first immunization. Tumor size was measured every three days for 22 days. Data are shown as the mean ± standard error for 5 mice. (**c**) Luminescence from the tumors was measured using IVIS, and the total flux was quantified. (**d**) Effector cells were collected 9 days after the final immunization and incubated with CFSE-labeled target cells at an effector-to-target (E:T) ratio of 40:1 for 16 h. Specific lysis was calculated using the following formula: Specific Lysis (%) = [1 − (% CFSE^high^/% CFSE^low^)] × 100. Data from three independent experiments are presented as mean ± standard error. (**e**) Lymphocytes were collected from inguinal lymph nodes 9 days after the last immunization, stained with CFSE, and co-cultured with PBS or OLFM4 for two days. Lymphocyte proliferation was assessed by measuring the CFSE dilution. Representative data from three independent experiments are shown. (**f**) The number of IFN-γ-producing splenocytes was compared using ELISPOT after stimulation with PBS or OLFM4 for two days. * Statistically significant at *p* < 0.05, according to Student’s *t*-test.

We evaluated tumor growth in mice following treatment with M-HIFU and S.C.-injected DCs preloaded with peptides (DCs[SC]). B6 mice were injected with B16F10-Luc2-OLFM4 cells in their right flanks and treated with either M-HIFU or DCs[SC] three times, as indicated (▼) (Figure 2a). Mice that received a combination of DCs[SC] and M-HIFU exhibited significantly greater tumor growth reduction than those treated with either DCs[SC] or M-HIFU alone, indicating the enhanced therapeutic effect of the combined treatment (Figure 2b). Luciferase signals emitted from the tumor cells were measured using the IVIS system (Figure 2c). Treatment with the combination of DCs[SC] and M-HIFU resulted in a remarkable 93.9 ± 8.28% inhibition of tumor growth compared to the N.C. group. In contrast, tumor reductions of 76.09 ± 19.98% for DCs[SC] and 49.2 ± 23.39% for M-HIFU alone were observed compared to the N.C. group (Figure 2c).

After treatment, the cytotoxicity of OLFM4-specific CTLs was assessed. OLFM4-positive target cells and OLFM4-negative mock cells were stained with 5 μM CFSE (CFSE^high^) and 0.5 μM CFSE (CFSE^low^), respectively (Figure S1). In the subcutaneous tumor model, lymphocytes from mice treated with the combination of DCs[SC] and M-HIFU demonstrated enhanced lysis of OLFM4-positive target cells, which was further amplified by combined treatment with DCs[SC] (Figure 2d). The percentages of target cell-specific lysis by CTLs at effector-to-target ratios of 10:1, 20:1, and 40:1 were 25.7 ± 7.8%, 43.5 ± 9.5%, and 66.6 ± 9.3%, respectively (Figure 2d).

Lymphocytes from inguinal lymph nodes harvested on day 22 were analyzed for OLFM4-specific proliferation after CFSE staining. CD8^+^ T cells from mice treated with DCs[SC] or DCs[SC] + M-HIFU showed strong proliferation in response to OLFM4 in vitro compared to those treated with M-HIFU or N.C. However, the combination of M-HIFU and DCs[SC] did not lead to significantly greater proliferation than DCs[SC] alone (Figure 2e). To further assess T-cell activity, the number of IFN-γ-producing splenocytes was measured using ELISPOT. The DCs[SC] + M-HIFU group exhibited a significant increase in OLFM4-specific IFN-γ-producing splenocytes compared to the DCs[SC], M-HIFU, and N.C. groups when stimulated with either PBS or recombinant OLFM4 protein (Figure 2f). These results suggest that M-HIFU induces a stronger OLFM4-specific immune response compared to T-HIFU in tumor-bearing mice. The enhanced anti-tumor effects observed with M-HIFU, including increased OLFM4-specific cytotoxic lymphocyte activity and a higher frequency of IFN-γ-producing splenocytes, indicate its potential as a more effective approach for targeting OLFM4-expressing tumors. These findings indicate that the combined treatment with M-HIFU and DCs[SC] leads to a stronger OLFM4-specific immune response and more pronounced tumor suppression in OLFM4-expressing tumor models. The observed increase in cytotoxic activity and the higher number of IFN-γ-producing splenocytes emphasize the effectiveness of this combined approach as a therapeutic option for targeting OLFM4-expressing tumors.

### 3.3. M-HIFU and DC Vaccine Administration Affects DC Migration and Tumor Microenvironment (TME)

**Figure 3:**
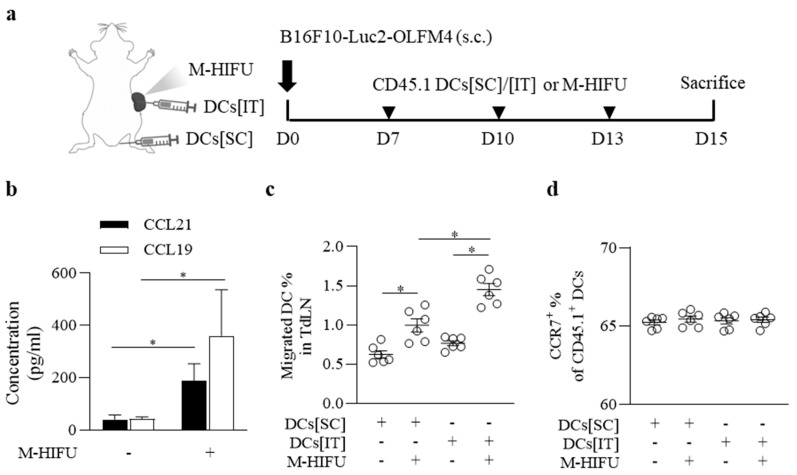
M-HIFU and DC vaccine administration effects on DC migration and tumor microenvironment (TME). (**a**) Schematic representation of the experimental design. CD45.1^+^ DCs were injected either intratumorally (I.T.) or subcutaneously (S.C.) after M-HIFU treatment (▼). The migration of DCs to the lymph nodes and chemokine expression in the tumor microenvironment (TME) were evaluated. (**b**) Chemokine expression (CCL19, CCL21) at the tumor site was measured using quantitative RT-PCR. M-HIFU significantly increased the expression of both CCL19 and CCL21 compared to the control, suggesting alterations in the TME. The experiments were conducted in duplicate, with a total of n = 16 per group (*p* < 0.01). (**c**) Quantification of migrated DCs in the lymph nodes after treatment. M-HIFU significantly enhanced DC migration compared to the control (n = 6 per group, *p* < 0.05). Migrated DC percentage = (percentage of CD45.1^+^ × Total lymph node cell number)/Total number of injected cells × 100 (**d**) CCR7 expression levels in the migrated DCs were assessed using flow cytometry. No significant differences in CCR7 expression were observed between the treatment and control groups (n.s., not significant). * Statistically significant at *p* < 0.05, according to Student’s *t*-test.

The potential of HIFU to induce significant changes in the tumor microenvironment (TME) has been highlighted in several studies. These studies demonstrated that HIFU can enhance immune cell infiltration and increase the expression of immune-regulating cytokines, contributing to improved immune responses against tumors. Based on these previous findings, we sought to evaluate how the combination of M-HIFU and DC vaccine administration affects DC migration and modulates the TME. To examine the effects of M-HIFU and dendritic cell (DC) vaccine administration on DC migration and the tumor microenvironment (TME), CD45.1^+^ mice were used, and DC migration through the lymph nodes (LNs) was measured. The schematic diagram (Figure 3a) shows the experimental design, highlighting the administration of M-HIFU and DC vaccine via intratumoral injection (DCs[IT]) or subcutaneous injection (DCs[SC]). After three treatments were administered every three days, sampling and analysis were performed two days after the final treatment (Figure 3a), revealing that M-HIFU significantly affected the TME. Notably, there was a significant increase in the expression of the chemokines CCL19 and CCL21 at the tumor site, especially in the M-HIFU-treated group. This elevated chemokine expression was associated with an altered TME, which may enhance DC recruitment and potentially boost anti-tumor immune responses (Figure 3b). Migrated DCs (Figure S4) were quantified in the lymph nodes (LNs), showing significant differences between the M-HIFU-treated and untreated groups in both DCs[SC] and DCs[IT] groups. Although M-HIFU treatment enhanced DC migration in the DCs[SC] group, the effect of M-HIFU was more pronounced in the DCs[IT] group, with a greater increase in DC migration observed in this group (Figure 3c). To evaluate the functionality of the migrated DCs, CCR7 expression was analyzed. However, CCR7 levels did not differ significantly between the groups (Figure 3d), suggesting that the success of DC migration induced by the DC vaccine is less dependent on CCR7 expression and more influenced by the impact of M-HIFU on the cytokine environment within the TME.

### 3.4. Comparative Efficacy of Subcutaneous vs. Intratumoral DC Vaccination in Conjunction with Mechanical HIFU in Tumor Models

**Figure 4:**
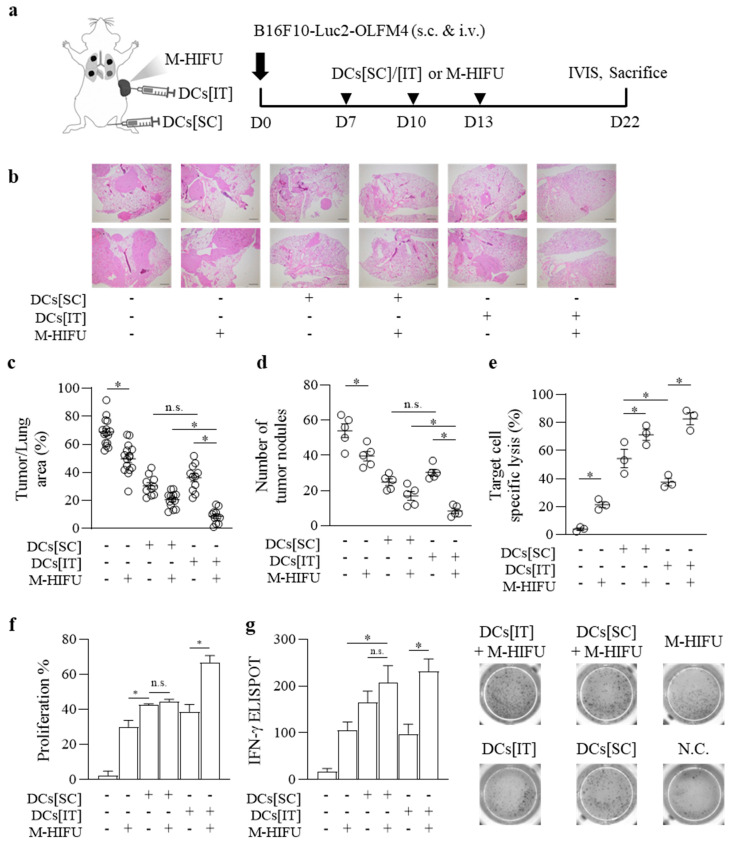
Comparative efficacy of subcutaneous vs. intratumoral DC vaccination in conjunction with mechanical HIFU in tumor models. (**a**) B16F10 cells were injected both subcutaneously (S.C.) and intravenously to induce cancer in both the subcutaneous region and the lungs, establishing a model of lung metastasis along with S.C. tumors. M-HIFU was applied to the S.C. tumor site, and dendritic cells (DCs) were administered either subcutaneously at the base of the tail or intratumorally (▼). (**b**) After the mice were sacrificed, lung paraffin blocks were prepared, and histological sections were stained with H&E. (**c**) The percentage of the tumor area relative to the total lung area was quantified using ImageJ software V2 by measuring the cancerous regions and the overall lung area from the histological sections. (**d**) Additionally, the number of visible tumor nodules in the lungs was manually counted. (**e**) Effector cells were harvested 9 days after the final immunization and incubated with CFSE-labeled target cells at an effector-to-target (E:T) ratio of 40:1 for 16 h. Specific lysis was calculated using the formula Specific Lysis (%) = [1 − (% CFSE^high^/% CFSE^low^)] × 100. Data from three independent experiments are presented as mean ± standard error. (**f**) Lymphocytes were collected from inguinal lymph nodes 9 days after the final immunization, stained with CFSE, and co-cultured with PBS or OLFM4 for two days. Lymphocyte proliferation was assessed by measuring CFSE dilution. Data from three independent experiments are presented. (**g**) The number of IFN-γ-producing splenocytes was compared using ELISPOT after two days of stimulation with OLFM4. * Statistically significant at *p* < 0.05, according to Student’s *t*-test.

B16F10 melanoma cells were injected S.C. and I.T. into mice to induce S.C. tumors and lung metastases. M-HIFU treatment was applied to S.C. tumors, followed by the administration of DCs either subcutaneously (DCs[SC]) or intratumorally (DCs[IT]). Mice received M-HIFU, DCs[SC], or DCs[IT] treatment three times, as indicated by the arrows (▼) (Figure 4a). Histological analysis of H&E-stained lung sections revealed metastatic tumor cell infiltration in the lung tissue, showing varying tumor burdens between the groups treated with DCs[SC] or DCs[IT] combined with M-HIFU. Some sections showed more extensive tumor spread, whereas others showed reduced metastasis, depending on the treatment method (Figure 4b).

The tumor area relative to the total lung area was quantified using the ImageJ software, and the graphs displayed the tumor burden. The results demonstrated a significantly smaller tumor area in the lungs of the DCs[IT] group compared to the DCs[SC] group, suggesting that the combination of DCs[IT] with M-HIFU more effectively reduced lung metastasis (Figure 4c). The number of visible tumor nodules in the lungs was also counted, showing a significant reduction in nodules in the DCs[IT] + M-HIFU group compared to that in the DCs[IT] group, further supporting the hypothesis that M-HIFU enhances anti-tumor efficacy in this model (Figure 4d).

Effector cells were harvested and incubated with target cells at a 40:1 effector-to-target (E:T) ratio to evaluate cytotoxicity. The graph revealed a higher percentage of specific lysis in the DCs[IT] group compared to the DCs[SC] group, indicating a stronger immune response to intratumoral DC administration in combination with M-HIFU (Figure 4e).

Additionally, a lymphocyte proliferation assay was performed, in which lymphocytes from inguinal lymph nodes were stained with CFSE and co-cultured with OLFM4. The results showed a greater lymphocyte proliferation rate, as indicated by CFSE dilution, in the DCs[IT] + M-HIFU group, suggesting enhanced T-cell activation and proliferation compared to that in the DCs[IT] group (Figure 4f).

IFN-γ production was assessed using ELISPOT after stimulating the splenocytes with PBS or OLFM4. The chart highlights a significantly higher number of IFN-γ-producing cells in the DCs[IT] + M-HIFU group (Figure 4g). Overall, both DCs[SC] and DCs[IT] were affected by M-HIFU; however, the DCs[IT] + M-HIFU group showed the most effective activation. Among all the groups, DCs[IT] was more significantly affected by M-HIFU.

Overall, both DCs[SC] and DCs[IT] treatments were significantly influenced by M-HIFU; however, the combination of DCs[IT] and M-HIFU showed the greatest reduction in lung metastasis and the highest level of immune activation. These results indicate that the intratumoral administration of DCs in conjunction with M-HIFU may be a more effective strategy for suppressing tumor growth and metastasis.

## 4. Discussion

OLFM4, a glycoprotein belonging to the olfactomedin family, is expressed in stem cells located at the base of intestinal crypts [29] and is associated with colorectal cancers through its colocalization with the G-protein-coupled receptor 5 (Lgr5) on columnar stem cells in the crypt base [30]. These Lgr5+ crypt base cells exhibit radiation resistance and do not retain DNA labels, distinguishing them from +4 position cryptic cells [31]. OLFM4 has been proposed to function as an “inducible resistance factor” against apoptotic triggers such as radiation and chemotherapy, highlighting its potential as a therapeutic target in cancer treatment [5].

Interestingly, OLFM4 expression has been detected in a variety of cancers, including those of the colon, breast, lung, pancreas, stomach, prostate, colorectum, liver, ovary, and cervix. However, its functional role appears to vary depending on the cancer type and context. In mouse melanoma cell lines, OLFM4 has been shown to suppress cancer growth and metastatic potential by downregulating integrin and MMP genes, suggesting a tumor-suppressive function in certain cancers [9]. Conversely, in human colorectal cancer (CRC), OLFM4 primarily acts as a differentiation marker rather than a direct driver of carcinogenesis or metastasis [32]. These findings underscore the context-dependent role of OLFM4 in tumorigenesis, positioning it as a promising yet nuanced target for cancer therapy, warranting further investigation into its mechanistic effects in different cancer types.

T-HIFU and M-HIFU both contribute to tumor ablation, but their immunological effects differ significantly due to their distinct mechanisms of action. T-HIFU induces coagulative necrosis through localized heating, leading to the controlled release of tumor antigens. However, the intense thermal environment can also suppress immune responses by damaging immune cells and altering the TME to favor immunosuppression. In contrast, M-HIFU promotes a more robust immunological response through mechanical disruption and cavitation effects. These mechanical forces induce the release of damage-associated molecular patterns (DAMPs), pro-inflammatory cytokines, and a broader spectrum of tumor antigens, which enhance dendritic cell (DC) activation and maturation.

Notably, M-HIFU plays a critical role in modulating the TME to enhance the efficacy of DC vaccines. By increasing the expression of migration factors such as CCL19 and CCL21, M-HIFU improves the migratory capacity of DCs, facilitating their movement to lymph nodes. This promotes effective antigen presentation and strengthens the activation of tumor-specific T cells [15]. These features highlight the unique advantages of M-HIFU over T-HIFU in amplifying immune responses, making it a promising adjunct to immunotherapies such as DC vaccines.

Despite these promising effects of HIFU, there are still limitations that need to be addressed through further research [33]. M-HIFU and T-HIFU face challenges in treating distant tumors, including difficulties in accurately focusing ultrasound on deep or critical areas [34], energy absorption or scattering that reduces efficacy [35], and risks of damaging adjacent tissues [36]. The complex tumor microenvironment also affects treatment response, with incomplete ablation increasing recurrence risk. Advancements in imaging, drug delivery integration, and microenvironment research are needed to overcome these challenges [37].

Several studies have shown that the CCL19/CCL21-CCR7 axis is crucial for promoting the infiltration of immune cells, including DCs and T cells, into tumor sites. The increased migration and survival of immune cells in the tumor microenvironment (TME), driven by CCL19 and CCL21, are linked to improved therapeutic outcomes, especially when combined with immunotherapies [38,39]. Additionally, experiments using engineered CAR-T cells co-expressing IL-7 and CCL21 demonstrated enhanced anti-tumor efficacy, attributed to the increased survival and infiltration of both CAR-T cells and DCs [40]. These findings suggest that strategies enhancing the CCL19/CCL21 pathway, possibly through M-HIFU, could potentiate immune responses against tumors by increasing DC migration and activation.

Both CCL19 and CCL21 interact with the CCR7 receptor on DCs, promoting their migration to lymph nodes and other immune-active regions. This migration is crucial for enhancing antigen presentation and stimulating adaptive immune responses within the TME [41]. In tumor-targeting approaches like M-HIFU, local stress and inflammation from focused ultrasound can increase the release of these chemokines, aiding DC influx and strengthening the anti-tumor immune response [42]. Notably, there was no difference in CCR7 expression levels on DCs, indicating the changes in chemokines CCL19/21 within the TME. The variation in migration effects was solely due to differences in the influence of HIFU CCL19 and CCL21.

M-HIFU and T-HIFU impact the TME differently, which can influence their effectiveness when combined with immunotherapies such as DC vaccines. M-HIFU primarily disrupts tumor tissue through mechanical forces, creating cavitation bubbles that damage tumor structures and promote the release of tumor-associated antigens into the surrounding environment [43]. This mechanical action creates an inflammatory environment that recruits immune cells, including DCs, which play a crucial role in initiating immune responses. M-HIFU creates a TME that promotes DC migration by enhancing the expression of chemokines such as CCL19 and CCL21 [15]. This improved environment facilitates the movement of DCs, particularly those delivered via intratumoral injection, toward the lymph nodes, where they can more effectively initiate an immune response. In contrast, DCs introduced through tail-based S.C. injection seemed to benefit less from the functional effects of HIFU, indicating that the delivery method may influence the efficacy of HIFU in boosting DC migration (Figure 3). Once exposed to the TME, DCs activate and migrate to lymph nodes, presenting antigens to T cells to initiate a strong adaptive immune response [43,44].

M-HIFU has a unique mechanism that enhances immune responses by physically disrupting tumor cells, releasing a broad range of antigens, and activating antigen-presenting cells (APCs). This distinguishes M-HIFU from other immunotherapies, such as checkpoint inhibitors that enhance T-cell function, cancer vaccines targeting specific antigens, and CAR-T cell therapies with high target specificity. Compared to intratumoral injections, which induce localized immune responses, M-HIFU demonstrates superior efficacy by facilitating extensive antigen release. Notably, M-HIFU can overcome tumor heterogeneity and amplify the initial immune response, making it a promising complementary approach to other immunotherapies.

These characteristics are particularly compelling in combination with PD-1/PD-L1 blockade or CAR-T cell therapies. M-HIFU induces tumor antigen release without extensive heat exposure, creating a microenvironment that facilitates an anti-tumoral response. Instead, it enhances immune cell infiltration and promotes the secretion of pro-inflammatory cytokines, shaping a tumor microenvironment conducive to CTL activation and improving the efficacy of immune checkpoint inhibitors [14,45]. Additionally, the immunomodulatory effects of M-HIFU may synergize with CAR-T cell therapy by enhancing CAR-T cell infiltration and persistence within the tumor microenvironment. The mechanical disruption caused by M-HIFU could facilitate CAR-T cell trafficking and its ability to overcome physical and immunosuppressive barriers in the tumor [46]. These synergistic effects suggest that combining M-HIFU with contemporary immunotherapies such as PD-1/PD-L1 blockade or CAR-T cell therapy could significantly improve therapeutic efficacy in highly aggressive and treatment-resistant tumors.

In contrast, T-HIFU affects tumor tissues by generating heat that ablates the tissue and induces thermal necrosis. While this process also releases tumor antigens, the heat exposure can foster the TME. For instance, T-HIFU increases the expression of inhibitory molecules like PD-L1, which suppresses CTL activation and weakens the overall immune response [16]. This immunosuppressive effect limits T-HIFU’s potential in combination therapies, particularly with immunotherapies like DC vaccines [44].

When combined with DC vaccines, M-HIFU proves significantly more effective than T-HIFU alone. The mechanical disruption caused by M-HIFU not only releases a higher quantity of tumor antigens but also triggers stronger immune activation. This physical destruction of tumor tissue enhances antigen presentation by DCs, which can then migrate more effectively to the lymph nodes to activate T cells. This activation fosters the proliferation of CTLs and natural killer cells, both crucial for targeting and destroying tumor cells [47]. In addition, the localized inflammatory response generated by M-HIFU increases the recruitment and maturation of dendritic cells, further amplifying the ability of the immune system to recognize and attack tumor cells. This synergy between M-HIFU and DC vaccines results in a more robust and sustained anti-tumor immune response, which is less likely to occur with T-HIFU, owing to its thermal nature and potential to induce immunosuppressive conditions [15].

## 5. Conclusions

The combination therapy of M-HIFU and DC vaccines demonstrates significant potential for the treatment of metastatic tumors by eliminating primary tumors through M-HIFU and suppressing metastatic growth with DC vaccines. This strategy enhances immune responses by boosting DC activation and shows superior tumor reduction effects compared to T-HIFU. To advance this approach to clinical trials, it is essential to conduct safety evaluations and optimize M-HIFU parameters as well as the dosage and timing of DC vaccine administration. The efficacy should be validated across various cancer types, with a focus on modulating the tumor microenvironment (TME) and targeting immunosuppressive pathways such as PD-L1 and TGF-β. Exploring synergies with modern immunotherapies, such as checkpoint inhibitors or CAR-T cell therapy, and supporting these findings with preclinical models is crucial. These efforts will contribute to the development of M-HIFU as a more precise and effective cancer treatment strategy [48].

## Data Availability

The raw data supporting the conclusions of this article may be made available by the authors on request.

## Supplementary Materials

The following supporting information can be downloaded at: https://www.mdpi.com/article/10.3390/pharmaceutics17010065/s1, Figure S1: Gating strategy and representative FACS plots for target cells stained with CFSE. B16F10 mock cells (CFSE^low^) and OLFM4-expressing B16F10 cells (CFSE^high^) were stained with different concentrations of CFSE. Effector cells (lymphocytes from immunized mice) were not labeled with CFSE (CFSE^−^). The CFSE^high^ and CFSE^low^ populations were analyzed to assess specific lysis of OLFM4-expressing cells by comparing the reduction in the CFSE^high^ population relative to the CFSE^low^ population; Figure S2: Gating strategy for analyzing dendritic cell (DC) differentiation. DAPI^−^ singlet cells were gated to focus on CD11b^+^ cells. CD11b^+^ F4/80^−^ CD11c^+^ cells were subsequently gated and analyzed to identify DCs; Figure S3: Gating strategy for analyzing CD8^+^ T cell proliferation using flow cytometry. Single-cell suspensions from inguinal and periaortic lymph nodes were stained and analyzed. DAPI^−^ singlet cells were gated to isolate CD8^+^ T cells, and their proliferation was evaluated by measuring CFSE dilution; Figure S4: Gating strategy for analyzing CD45.1^+^ cells and their CCR7^+^ expression using flow cytometry. Single-cell suspensions from inguinal and periaortic lymph nodes were stained and analyzed by flow cytometry. DAPI^−^ singlet cells were gated to identify CD45.1^+^ and CD45.2^+^ populations, with CCR7^+^ expression subsequently evaluated within the CD45.1^+^ cell population; Figure S5: Confocal microscopy analysis of dendritic cells (DCs) transduced with PBS (upper panel) or 50 μg/mL recombinant P-OLFM4 (lower panel). DCs were stained with anti-OLFM4-Alexa Fluor 546 (red) and DAPI (blue), highlighting Alexa Fluor 546^+^ DAPI^+^ cells. Scale bar: 50 µm.
