# Supplementary material for: HIFU-CCL19/21 Axis Enhances Dendritic Cell Vaccine Efficacy in the Tumor Microenvironment
Source: Pharmaceutics. 2025 Jan 6;17(1):65. doi: 10.3390/pharmaceutics17010065 (PMC11769570; doi:10.3390/pharmaceutics17010065)
Supplement: Supplementary file 1 [file pharmaceutics-17-00065-s001.zip › pharmaceutics-3357372-supplementary.pdf]

pharmaceutics

Article

HIFU-CCL19/21 Axis Enhances Dendritic Cell Vaccine Efficacy in the Tumor Microenvironment

Bum-Seo Baek, Hyunmi Park, Ji-Woong Choi, Eun-Young Lee and Seung-Yong Seong *

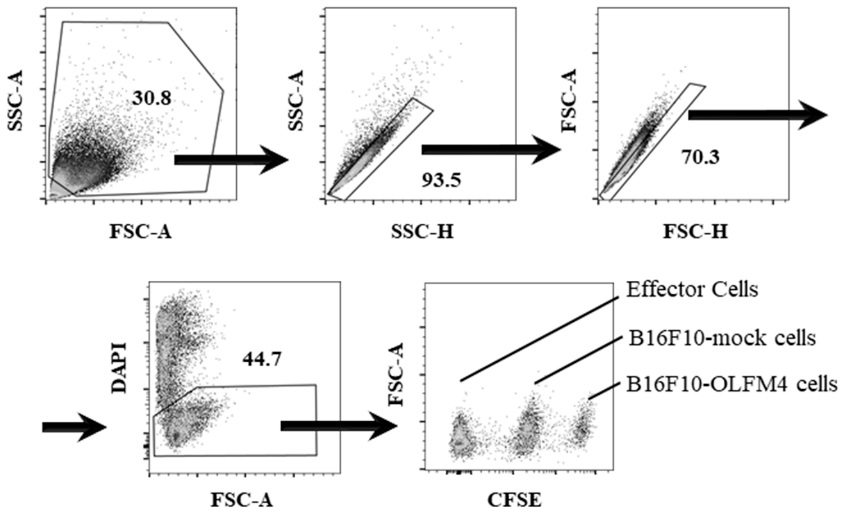

Figure S1. Gating strategy and representative FACS plots for target cells stained with CFSE. B16F10 mock cells (CFSE^{low}) and OLFM4-expressing B16F10 cells (CFSE^{high}) were stained with different concentrations of CFSE. Effector cells (lymphocytes from immunized mice) were not labeled with CFSE (CFSE^{-}). The CFSE^{high} and CFSE^{low} populations were analyzed to assess specific lysis of OLFM4-expressing cells by comparing the reduction in the CFSE^{high} population relative to the CFSE^{low} population.

Academic Editors: Guangsheng Du and Lian Li

Received: 21 November 2024
Revised: 26 December 2024
Accepted: 3 January 2025
Published: 6 January 2025

Citation: Baek, B.-S.; Park, H.; Choi, J.-W.; Lee, E.-Y.; Seong, S.-Y. HIFU-CCL19/21 Axis Enhances Dendritic Cell Vaccine Efficacy in the Tumor Microenvironment. *Pharmaceutics* **2025**, *17*, 65. https://doi.org/10.3390/pharmaceutics17010065

Copyright: © 2025 by the authors. Licensee MDPI, Basel, Switzerland. This article is an open access article distributed under the terms and conditions of the Creative Commons Attribution (CC BY) license (https://creativecommons.org/licenses/by/4.0/).

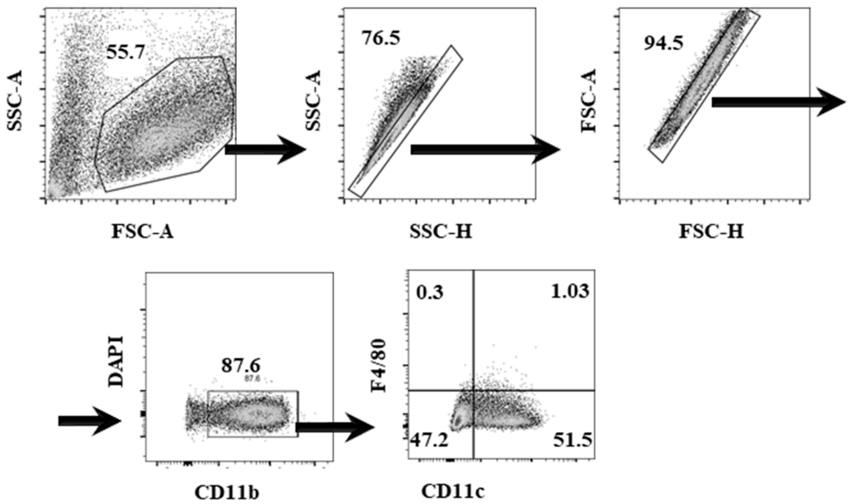

Figure S2. Gating strategy for analyzing dendritic cell (DC) differentiation. DAPI- singlet cells were gated to focus on CD11b^{+} cells. CD11b^{+} F4/80^{-} CD11c^{+} cells were subsequently gated and analyzed to identify DCs.

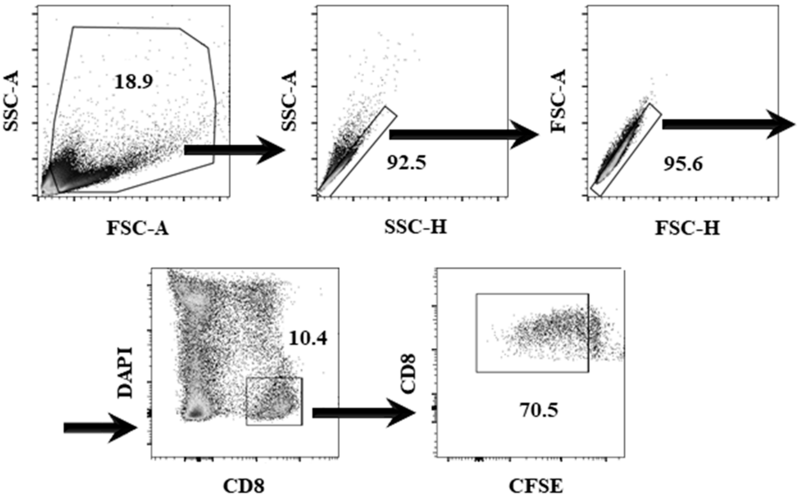

Figure S3. Gating strategy for analyzing CD8⁺ T cell proliferation using flow cytometry. Single-cell suspensions from inguinal and periaortic lymph nodes were stained and analyzed. DAPI⁻ singlet cells were gated to isolate CD8⁺ T cells, and their proliferation was evaluated by measuring CFSE dilution.

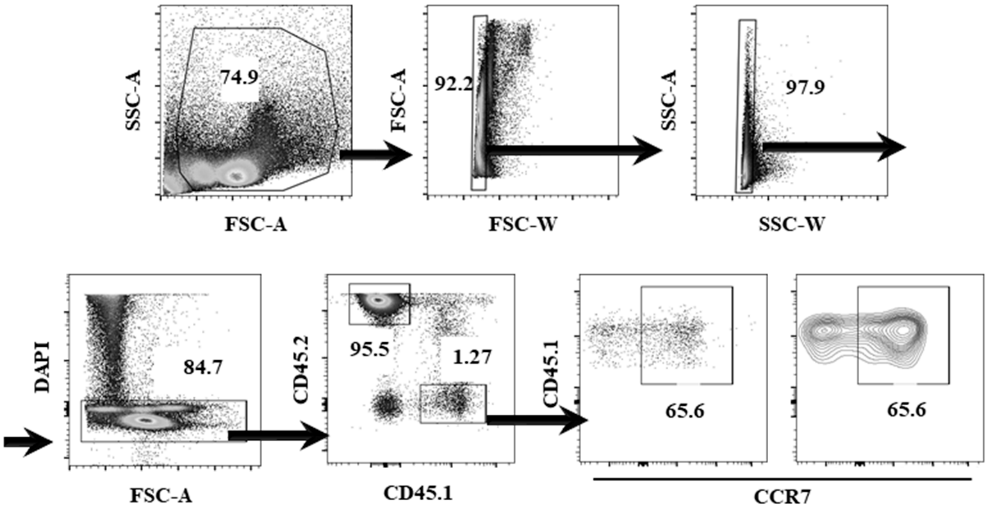

Figure S4. Gating strategy for analyzing CD45.1⁺ cells and their CCR7⁺ expression using flow cytometry. Single-cell suspensions from inguinal and periaortic lymph nodes were stained and analyzed by flow cytometry. DAPI⁻ singlet cells were gated to identify CD45.1⁺ and CD45.2⁺ populations, with CCR7⁺ expression subsequently evaluated within the CD45.1⁺ cell population.

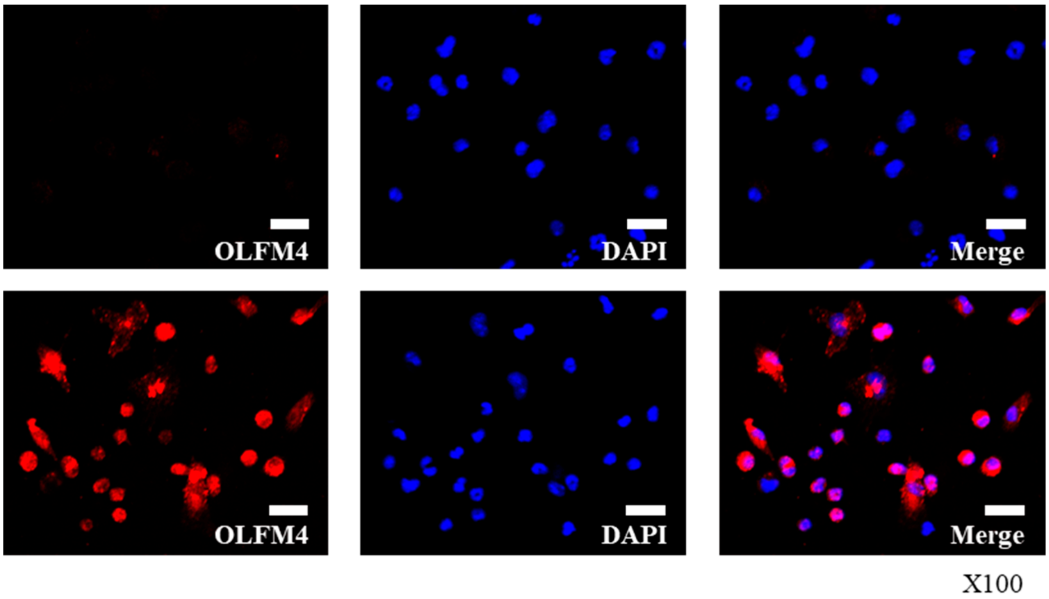

Figure S5. Confocal microscopy analysis of dendritic cells (DCs) transduced with PBS (upper panel) or 50 µg/mL recombinant P-OLFM4 (lower panel). DCs were stained with anti-OLFM4-Alexa Fluor 546 (red) and DAPI (blue), highlighting Alexa Fluor 546^{+} DAPI^{+} cells. Scale bar: 50 µm.
